# Segmental Maternal UPD of Chromosome 7q in a Patient With Pendred and Silver Russell Syndromes-Like Features

**DOI:** 10.3389/fgene.2018.00600

**Published:** 2018-11-30

**Authors:** Valentina Cirello, Valentina Giorgini, Chiara Castronovo, Susan Marelli, Ester Mainini, Alessandra Sironi, Maria Paola Recalcati, Marco Pessina, Daniela Giardino, Lidia Larizza, Luca Persani, Palma Finelli, Silvia Russo, Laura Fugazzola

**Affiliations:** ^1^Division of Endocrine and Metabolic Diseases, Laboratory of Endocrine and Metabolic Research, IRCCS Istituto Auxologico Italiano, Milan, Italy; ^2^Laboratory of Medical Cytogenetics and Molecular Genetics, IRCCS Istituto Auxologico Italiano, Milan, Italy; ^3^Neuropsychiatry and Neurorehabilitation Unit, Scientific Institute, IRCCS Eugenio Medea, Lecco, Italy; ^4^Department of Medical Biotechnologies and Translational Medicine, University of Milan, Milan, Italy; ^5^Department of Clinical Sciences and Community Health, University of Milan, Milan, Italy; ^6^Department of Pathophysiology and Transplantation, University of Milan, Milan, Italy

**Keywords:** pendred syndrome, silver-russell syndrome, *SLC26A4*, post-natal growth retardation, uniparental disomy

## Abstract

Pendred syndrome (PS) is an autosomal recessive disorder due to mutations in the *SLC26A4* gene (chr7q22. 3) and characterized by sensorineural hearing loss and variable thyroid phenotype. Silver-Russell syndrome (SRS) is a heterogeneous imprinting disorder including severe intrauterine and postnatal growth retardation, and dysmorphic features. Maternal uniparental disomy of either the whole chromosome 7 (upd(7)mat) or 7q (upd(7q)mat) is one of the multiple mechanisms impacting the expression of imprinted genes in SRS, and is associated with milder clinical features. Here, we report genetic and clinical characterization of a female child with PS, postnatal growth retardation, and minor dysmorphic features. A gross homozygous deletion of *SLC26A4* exons 17-20 was suspected by Sanger sequencing and then confirmed by array-CGH. Moreover, an insertion of about 1 kb of the *CCDC126* gene (7p15.3), which does not appear to be clinically relevant, was detected. The possible occurrence of a balanced rearrangement between 7p and 7q was excluded. The absence of the deletion in the father led to the investigation of upd, and microsatellite segregation analysis revealed a segmental 7q (upd(7q)mat), leading to *SLC26A4* homozygosity and responsible for both PS and SRS-like traits. The proband matched 3 out of 6 major SRS criteria. In conclusion, this is the first report of uniparental isodisomy encompassing almost the whole long arm of chromosome 7 resulting in PS and SRS-like features. Whereas, the inner ear phenotype of PS is typical, the clinical features suggestive of SRS might have been overlooked.

## Introduction

Pendred syndrome (PS [MIM: 274600]) is an autosomal recessive disorder, characterized by the association of sensorineural hearing loss (SNHL), inner ear malformations, and a partial iodide organification defect leading to an extremely variable thyroid phenotype (Everett et al., 1997; Fugazzola et al., 2007). PS is due to an impaired function of pendrin, a transmembrane multifunctional anion exchanger encoded by the *SLC26A4* gene (MIM: 605646; chr7q22.3) (Everett et al., 1997), and mainly expressed at the inner ear, thyroid, and kidney levels (Everett et al., 1999; Bidart et al., 2000; Royaux et al., 2001). In the inner ear, pendrin functions as a chloride/bicarbonate exchanger crucial to the maintenance of the composition and the electrochemical potential of the endolymph (Everett et al., 1999). Its dysfunction results in the enlargement of the membranous labyrinth structures and the damage of the neuroepithelium secondary to osmotic and toxic mechanisms (Everett et al., 1999). The enlargement of the membranous labyrinth (endolymphatic duct and sac) and of the bony structures (vestibular aqueduct and cochlea) are documented in all cases (Fugazzola et al., 2000), as well as the congenital SNHL. Moreover, pendrin regulates iodide flux and bicarbonate secretion into the follicular lumen at the apical membrane of thyroid cells. Its impaired function leads to a partial iodide organification, which associates with a goiter of variable sizes and with subclinical hypothyroidism (Fugazzola et al., 2001). More than 400 different mutations of the *SLC26A4* gene have been described in PS patients, in compound heterozygosity or in homozygosity, or in non-syndromic autosomal recessive hearing loss (DFNB4 [MIM: 600791]). The vast majority of them involves a single nucleotide, while only 5 genomic gross deletions are reported in the human gene mutation database (http://www.hgmd.org/).

Occasionally, recessive disorders may occur as the consequence of duplication or uniparental disomy (upd) of a region including a heterozygous pathogenic variant. Chromosome 7 upd has been reported in patients with cystic fibrosis, primary ciliary dyskinesia, and osteogenesis imperfecta type III (Spotila et al., 1992; Bartoloni et al., 2002; Reboul et al., 2006). Complete or segmental upd(7)mat is also causative of 7–10% of Silver Russell syndrome (SRS [MIM: 180860]) cases. The majority of SRS cases are instead due to the loss of methylation (LOM) of 11p15 imprinting center region 1 (ICR1) domain (Eggermann, 2010). SRS is a clinically heterogeneous growth disorder whose diagnosis, according to the consensus statement (Netchine-Harbison clinical scoring system, NH-CSS), should include 4 out of 6 clinical criteria: intrauterine and postnatal growth retardation, relative macrocephaly, protruding forehead, body asymmetry, and severe feeding difficulties (Wakeling et al., 2017). Additional features include a typical facies, V finger clino- and brachidactyly, ear anomalies, and speech delay. The clinical features of whole or segmental upd(7)mat carriers are less characteristic than those of 11p15 LOM patients: the growth is less retarded, the morphological abnormalities are slight, whereas delayed development and speech are more common (Hannula et al., 2001a). The altered methylation of three differentially methylated regions, *GRB10*:alt-TSS-DMR, *PEG10*:TSS-DMR, and *MEST*:alt-TSS-DMR, is likely associated with the expression of the clinical features (Eggermann, 2010).

We report the first case of a female child with maternal segmental upd of chromosome 7 presenting with PS and SRS-like features.

## Case presentation

### Clinical report

**Figure 1:**
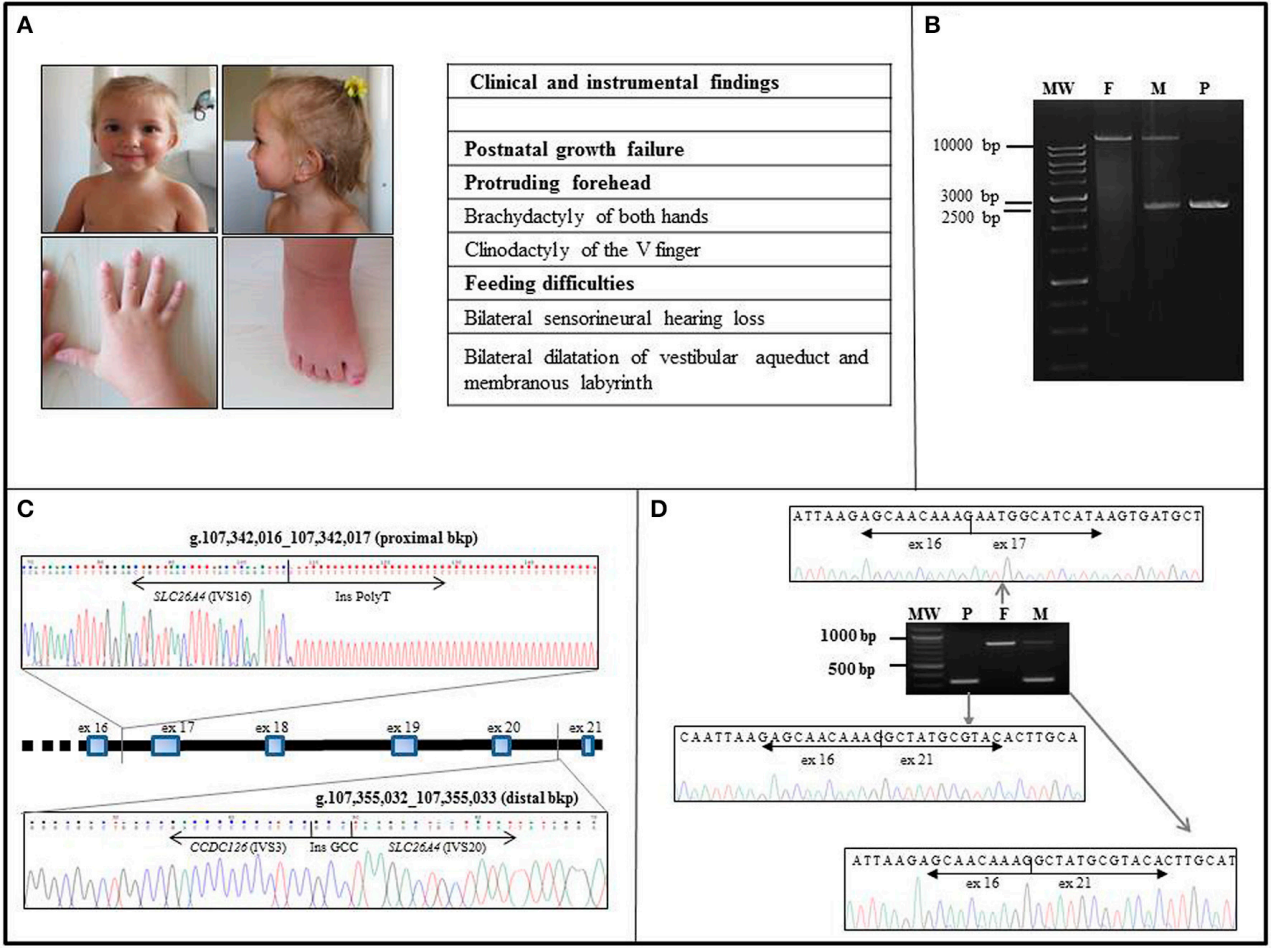
Clinical and molecular characterization of the case. **(A)** Frontal and lateral view of the proband at the age of 36 months. Note the mild frontal bossing, low-set posteriorly rotated ears, and thin lips. Earing aids are in place. The patient also displays brachydactyly of both hands and feet and clinodactily of V finger of hands. Other clinical findings are listed in the side table, where 3 out of 6 NH-CSS for SRS are in bold characters. **(B)** The LR-PCR amplicons of the patient (P) and both her father (F) and mother (M) were resolved on a 0.8% agarose gel. The patient (P) displayed a unique shorter band of about 2.5 kb in size, the father (F) showed a unique band of over 10 kb in size, corresponding to the expected 14.7 kb wild type band, whereas her mother (M) showed two bands corresponding to the wild type and the deleted alleles. MW, molecular weight. **(C)** The proximal and distal breakpoints of the *SLC26A4* intragenic deletion were mapped within *SLC26A4* IVS16 and the IVS20, respectively. The deletion is about 13 kb long. Sequence alignments of the junction fragments revealed an insertion of a part of *CCDC126* IVS3. The rejoining between *SLC26A4* IVS20 and *CCDC126* IVS3 distal bkp occurred through a de novo 3 bp GCC insertion. **(D)** Sequencing of the RT-PCR amplicons, extending from exons 13–14 to 3′UTR of *SLC26A4*, confirmed the homozygous deletion of exons 17–20 in the child (P). The father (F) showed only the long transcript corresponding to the wild type pendrin, whereas the mother (M) displayed a short transcript and a long one, consistent with a heterozygous state of the deletion. MW, molecular weight.

The proband is a 3 year-old girl born at 38 weeks by vaginal delivery after an uneventful pregnancy, second child of healthy non-consanguineous Caucasian parents with an uneventful family history. At birth, weight was 3,050 g (−0.09 SDS), length 49 cm (−0.1 SDS), and occipitofrontal circumference (OFC) 32.5 cm (−0.94 SDS). Neonatal SDSs were calculated according to the Italian Neonatal Study (INeS) charts (http://www.inescharts.com). Feeding difficulties and delayed growth were recorded during the perinatal period and first months of life. At 8 months (preverbal age), she was diagnosed with bilateral SNHL, and mutations in both *GJB2* and *GJB4* genes were ruled out. Magnetic resonance revealed a bilateral dilatation of both the vestibular aqueduct and the membranous labyrinth. Upon PS suspicion, appropriate genetic analysis was requested. At 26 months, weight was 9.2 kg (−2.09 SDS), height 79.5 cm (−2.51 SDS), and OFC 46.5 cm (−0.64 SDS), while at the last visit (34 months) weight 10.5 Kg (−2.09 SDS), height 86.5 cm (−2.20 SDS), and OFC was 47 cm (−0.98 SDS). Post-natal SDS were calculated according to the WHO Child Growth Standard (http://www.who.int/childgrowth/en/). Cranio-facial dysmorphic features included high forehead, mild frontal bossing, low-set posteriorly rotated ears, and thin lips. The patient also displayed brachydactyly of both hands and feet and clinodactyly of the V finger (Figure 1A). Thyroid function was normal, as found in most PS cases during infancy, as well as ophthalmological evaluation, heart and abdominal ultrasounds. Bone age corresponded to chronological age. Neuropsychiatric assessment showed a mild intellectual disability with expressive language delay. Neuropsychomotor evaluation at 34 months showed (Bayley scales): (a) cognitive scale: 25.15 months; (b) language scale: receptive communication subtest 14.15 months, expressive communication subtest 24.15 months; (c) motor scale: fine motor subtest 31.15 months, gross motor subtest 28.15 months. Social-emotional and adaptive behavior subscales were according to age. The study was approved by Ethical Clinical Research Committee of IRCCS Istituto Auxologico Italiano. Written informed consents to participate in the study and for publication were obtained from patients' parents.

## Methods

### Molecular and transcript analysis of *SLC26A4*

Genomic DNA (gDNA) and total RNA were extracted from whole blood samples of the patient and her parents with the Wizard Genomic DNA purification kit (Promega, Madison, USA) and the Tempus kit (ThermoFisher, Waltham, Massachusetts, USA), respectively, according to the manufacturer's instructions. The entire coding sequence and intron-exon junctions of the *SLC26A4* gene were amplified for mutation screening by PCR and direct sequencing, as previously reported (Cirello et al., 2012). To confirm the deletion and localize its breakpoints (bkp) at nucleotide level, long-range (LR) PCR spanning the *SLC26A4* genomic region from exon 16 to exon 21 was carried out with Takara LA Taq (Diatech, Jesi, Italy), according to cycle conditions suggested by the manufacturer. The following intronic primers were used: F 5′-TCTTTTTTGGCAGGATAGC-3′ and R 5′-TCGTCTGAATAATTCTAGCC-3′. The resulting LR-PCR products were sequenced and the sequences were aligned to the human reference genome sequence (human genome assembly GRCh37/hg19). cDNAs were obtained using the High-Capacity cDNA Reverse Transcription Kit (Thermofisher, Waltham, Massachusetts, USA) and RT-PCR was performed using the following primers: F 5′-GAGTTCAGTTTCCTTCTTGGA-3′ and R 5′-TCCCTTGCTCATAGAGACCTC-3′. The fragments obtained were purified and sequenced.

### Cytogenetic and molecular-cytogenetic analyses and characterization of CNV inheritance

High-resolution chromosomal Q-banding was performed on cultured peripheral blood lymphocytes of both the patient and her mother, according to standard cytogenetic procedures. For each sample at least 16 metaphases were analyzed. High-resolution array Comparative Genomic Hybridization (CGH) analysis was performed on genomic blood DNA of the patient and her mother, using the SurePrint G3 Human CGH Microarray 2 × 400K Kit (Agilent Technologies, Palo Alto, CA) following the manufacturer's protocol. Data were extracted and analyzed for copy number changes using Agilent CytoGenomics software v.3.0.6.6 (Agilent Technologies). Coordinates of Copy Number Variants (CNVs) referred to the Human Genome assembly GRCh37/hg19. CNVs classification was performed according to the Database of Genomic Variants (DGV) [(http://projects.tcag.ca/variation/) release: March 2016]. For the CNV not maternal in origin, inheritance was identified by performing quantitative PCR analysis on gDNA of both the proband and her parents using SYBR Green methodology. Two amplicons were chosen within non-repeated DNA segments using Primer3 software (http://bioinfo.ut.ee/primer3-0.4.0/):dupX_1F 5′-GAAGCCGTAGCAAGGAATGT-3′ and dupX_1R 5′-ATGGGAAAGCGACACAAATC-3′; dupX_2F 5′-GGAGGTGTTTCCTGGTGTGT-3′ and dupX_2R 5′-ACCGCCCTCAATCTCCAC-3′. A control amplicon was selected with the same parameters in the *PCNT* gene at 11q14.1 (PCNT-F: 5′-TCCAGAACATTCCTTGACAGAG-3′; PCNT-R: 5′-GTACCCCTCCCAATCTTTGC-3′). Amplification and detection were performed on ABI PRISM 7900HT Sequence Detection System (ThermoFisher Scientific, Waltham, MA). Each experiment was performed in triplicate on patient, parents and three controls known to not carry CNVs affecting the investigated locus. Relative quantification of the amount of DNA was obtained using the 2^−ΔΔ*Ct*^ method.

### Microsatellite analysis

**Figure 2:**
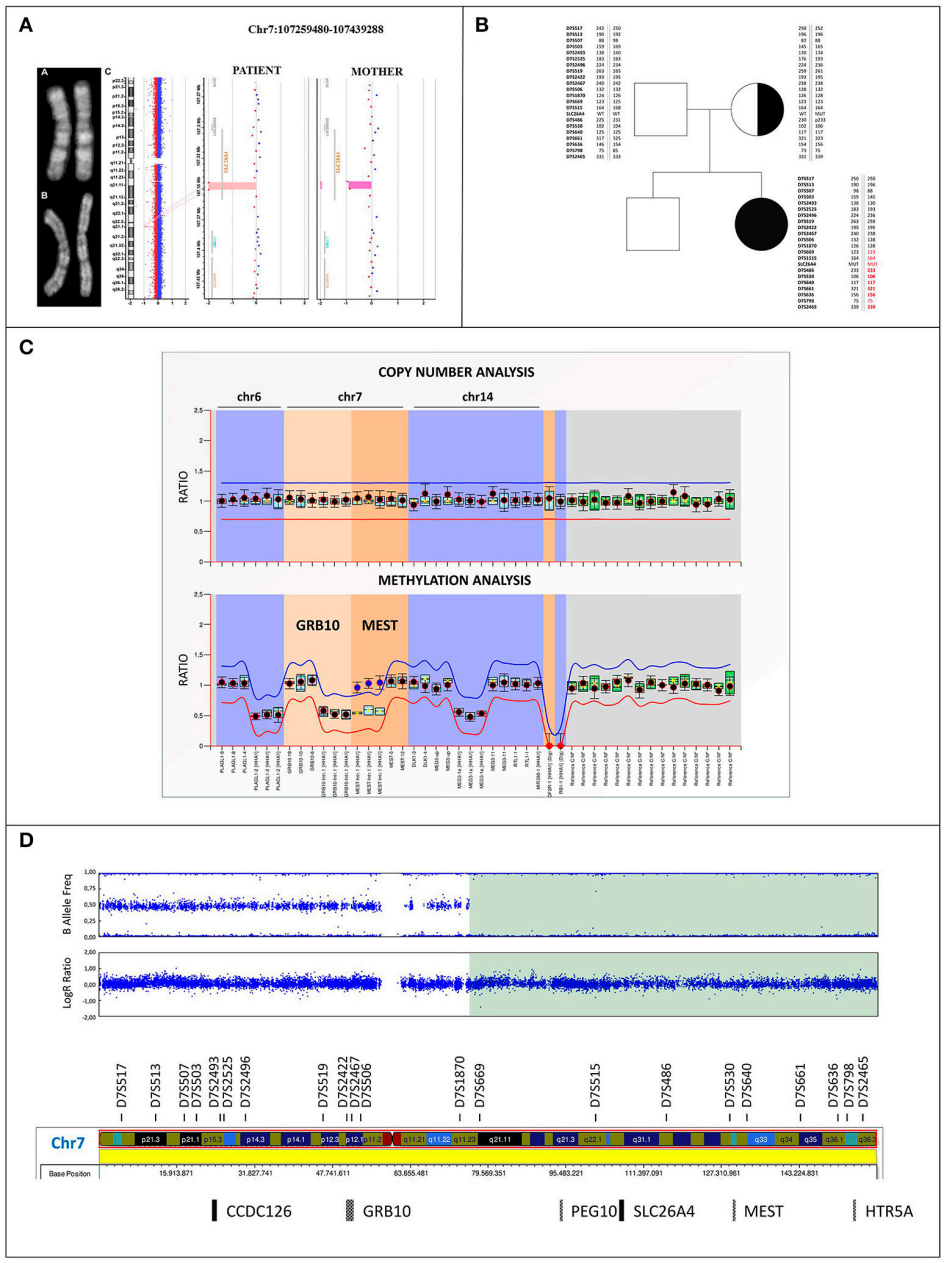
Cytogenetic and molecular characterization of upd(7)mat **(A)** Q-banding ruled out any apparently balanced structural rearrangement in the chromosome 7 homologous of both the patient **(a)** and her mother **(b)**. **(c)** Identification in the patient of a 6 kb homozygous deletion within the *SLC26A4* gene at 7q22.3 (minimum interval chr7:107344703_107350605, GRCh37/hg19) using Agilent CGH 400K array. The mother resulted heterozygous for the same deletion. **(B)** Family pedigree: filled symbol indicates the homozygous-affected proband and symbol with dot denotes carrier mother. Parents-to-proband segregation of alleles at 21 microsatellites spanning chromosome 7 is shown. STR markers mapping within the disomic region are highlighted in red, the markers showing the lack of paternal contribution are bolded. **(C)** MS-MLPA profile of the imprinted loci at chromosomes 6, 7, and 14 in the proband. Copy number quantification (top panel) and methylation ratio (bottom panel) for *GRB10*:alt-TSS-DMR and *MEST*:alt-TSS-DMR are shown. Each black dot displays the final probe ratio for each locus analyzed, and refers to the interval of values obtained by reference samples (light blue rectangles). The red and blue lines indicate the arbitrary borders for loss and gain, respectively. By default, the borders are placed ±0.3 from the mean probe value of a probe over the reference samples. No deviation from reference samples is observed for CNVs, indicating a biallelic contribution (top panel). The MS profile shows a gain of methylation (methylation ratio = 1) for the paternally imprinted *MEST*:alt-TSS-DMR in the proband, as shown by blue dots, with respect to reference samples (methylation ratio = 0.5). On the contrary, a proper methylation is observed at the *GRB10*:alt-TSS-DMR, as shown by black dots (bottom panel). Standard deviations were set up according to the Coffalyzer DB software v131211. **(D)** SNP array profile of patient chromosome 7. Top plot shows B allele frequency revealing an 83.5 Mb isodisomic region (7q11.23-qter) including *PEG10*:TSS-DMR, *MEST*:alt-TSS-DMR, and *HTR5A*:TSS-DMR imprinted loci; bottom plot shows Log R ratio, which reveals a proper biallelic contribution.

Microsatellite analysis was performed using 21 STR marker spanning the whole chromosome 7 as indicated in Figure 2B. All fluorescent PCR amplicons were genotyped on capillary electrophoresis using the 3,500 Genetic Analyzer (Applied Biosystems). Data analysis was carried on by the Genemapper software (Applied Biosystem) matching parental to proband transmission.

### Methylation-specific multiplex ligation-dependent probe amplification (MS-MLPA)

MS-MLPA (SALSA MLPA ME032 UPD7-UPD14, MRC Holland, The Netherlands), to investigate *GRB10* and *MEST* imprinted loci, was performed on the proband and reference controls (at least three references/each MLPA experiment), according to manufacturer's instructions. In detail, DNA was processed in parallel with and without digestion with the methylation sensitive HhaI enzyme to test the methylation deregulation and copy number variations (CNVs), respectively. Amplification products were processed by capillary electrophoresis using the 3,500 Applied Biosystems Genetic Analyzer and data analysis related to CNVs and methylation status was performed using the Coffalyzer DB software (Software version: v131211). The methylation status is defined for each single probe by the ratio of digested to undigested DNA, referring each test sample to control references.

### SNP array

Proband's DNA was genotyped by SNP array HumanCytoSNP-12 v2.1 BeadChip (Illumina INC, San Diego California, USA) using the BlueFuse Multi v.4.4 software (Illumina) and comparing log R ratio and B allelic frequency by the provided algorithm.

## Results

### *SLC26A4* molecular analysis

Starting from the patient's gDNA, we successfully amplified and found wild-type all *SLC26A4* exons, with the exception of exons 17-20. A large homozygous deletion was suspected, and LR-PCR spanning exons 16-21 showed a 14.7 kb single band, corresponding to the wild type product in the father, a single band of about 2.5 kb (consistent with the deletion of exons 17-20) in the child, and two bands of 14.7 and 2.5 kb in the mother (Figure 1B). We concluded that the proband was homozygous and the mother heterozygous for the deleted allele. Sequencing of the 2.5 kb LR-PCR product allowed the *SLC26A4* deletion bkps to be mapped at nucleotide level. Specifically, the proximal bkp is located within IVS16 at g.107342016_107342017 position, whereas the distal one within IVS20 at g.107355032_107355033 position (Figure 1C), resulting in a deletion of 13013 bases. Surprisingly, sequence alignment of the junction fragments revealed an insertion of about 1 kb of an unknown sequence identified as part of the IVS3 of the *CCDC126* (Coiled-Coil Domain Containing 126) gene, mapped at 7p15.3 (RefSeq Accession NM_138771) (Figure 1C). According to HGVS nomenclature (http://www.HGVS.org/varnomen), the identified genomic alteration is NC_000007.13(NM_000441.1):c.1804-255_2320-836delins [NC_000007.13 (NM_138771.3):c.239-5133_(239-4218_239-4177)inv;GCC].

### *SLC26A4* transcript analysis

Sequencing of the RT-PCR products confirmed the deletion of exons 17-20 in the child, without any aberrant splicing due to *CCDC126* IVS3 insertion. The detected short transcript corresponds to a truncated protein of 608 amino acids. The mother had a short and a long transcript, while the father had only the long transcript corresponding to the wt pendrin (Figure 1D).

### Conventional and molecular-cytogenetic analyses

A conventional cytogenetic analysis performed on the patient and her mother excluded a balanced complex structural chromosome aberration between p and q arms of chromosome 7, which might have mediated the del/ins rearrangement within *SLC26A4* (Figure 2A). Consistently, high resolution array CGH analysis did not identify in the proband any rare chromosome 7 CNV, except for the small homozygous deletion of about 6 kb at 7q22.3 (minimal interval chr7:107344703_107350605) of maternal origin affecting *SLC26A4*, according with the molecular results (Figure 2A). In addition, a rare duplication of 481 kb at Xq26.2 (minimum interval chrX:130693373_131174432) was detected, partially including *MST4* gene, which was not identified in the maternal genome. qPCR characterization showed the paternal origin of the duplication (Supplemental Figures 1A,B), excluding its clinical relevance.

### Microsatellite analysis

Microsatellites segregation across the whole chromosome 7 showed the absence of the paternal contribution of D7S486, D7S530, D7S640, D7S661, D7S636, D7S2465 markers, including the *MEST* gene, suggesting uniparental isodisomy and thus disclosing the homozygous expression of PS mutation (Figure 2B).

### MS-MPLA analysis

MS-MLPA evidenced a normal methylation ratio at the *GRB10*:alt-TSS-DMR, while an increased methylation value was observed at *MEST*:alt-TSS-DMR. Copy Number analysis revealed a normal *MEST* biallelic contribution, thus excluding the possible occurrence of a deletion, and indicating a deregulation of imprinting in the 7q region (Figure 2C).

### SNP array

Log ratio and B allelic frequency showed a segmental upd(7) spanning from 7q11.23 to qter, named arr[GRCh37] 7q11.23q36.3(75620641_159119486)x2 hmz mat according to the International System for Human Cytogenomic Nomenclature (ISCN 2016). The region includes the maternal mutated allele of *SLC26A4* gene, leading to absent pendrin expression (Figure 2D).

## Discussion

We report the first case of a female child with (PS) harboring a maternal segmental isodisomy of chromosome 7 (upd(7)mat). Upd may be causative of autosomal recessive diseases due to the loss of heterozygosity of recessive pathogenic variants, as reported for patients harboring a upd(7)mat and affected with cystic fibrosis (Reboul et al., 2006). In our case, the segmental upd(7)mat, encompassing almost the whole long arm of chromosome 7, led to the homozygosity of a 13 kb deletion in the *SLC26A4* allele of maternal origin. Sequencing of the aligned junction fragments revealed the insertion of about 1 kb of the IVS3 of the *CCDC126* gene, mapping to 7p15.3, which does not appear to be clinically relevant. The segmental isodisomy is probably due to a postzygotic mitotic recombination, occurring during the first zygotic division and followed by the loss of one daughter cell (Hannula et al., 2001b; Niida et al., 2018).

PS phenotype of our proband is classical, with congenital SNHL and the typical bilateral dilatation of the vestibular aqueduct. Interestingly, the occurrence of upd(7) and neurosensorial hearing loss, associated to inner ear malformations was previously reported, though no *SLC26A4* mutation were identified (Bigoni et al., in press). The patient here described presents with 3/6 NH-CSS criteria for SRS: postnatal growth retardation, prominent forehead, and feeding difficulties (Figure 1A). Additional features are borderline macrocephaly, low-set posteriorly rotated ears, brachydactyly of both hands, and feet, clinodactyly of the V finger, very mild syndactyly of toes, and high pitched voice. Moreover, mild intellectual disability with expressive language delay was diagnosed. Consistently, the analysis of the clinical features of six patients with upd(7q)mat previously reported reveals that only 2 of them fulfill all the NH-CSS consensus criteria (Table 1) (Hannula et al., 2001b; Reboul et al., 2006; Eggermann et al., 2008; Su et al., 2017). Severe postnatal growth delay and absence of body asymmetry are shared with complete upd(7)mat in all cases, and 6/7 cases, including the present, show postnatal relative macrocephaly (Table 1). Among additional SRS signs, triangular face, clinodactyly, and ears anomalies are the most represented (Table 2).

**Table 1:** Comparative overview of the clinical findings, according to the NH-CSS criteria, in the present case and in the six reported cases with segmental upd(7q)mat.

**NH-CSS CRITERIA**																		
	**Sex**	**Disomic region extention**	**GA weeks**	**IUGR**	**SGA**	**Birth**				**PNGF**	**At evaluation**								**NH-CSS score**
						**Weight gr (SD)**	**Length cm (SD)**	**OFC cm (SD)**	**Macro cephaly**	**PNGF**	**Age years**	**Weight gr (SD)**	**Height cm (SD)**	**OFC cm (SD)**	**Macro cephaly**	**Protruding forehead**	**Body asym**	**Feeding difficulties**	
Present case	F	7q11.23-qter	38	–	−	3.050 (−0.09)	49 (−0.1)	32.5 (−0.94)	–	+	2.1	9.200 (−2.09)	79.5 (−2.5)	46.5 (−0.64)	+/–	+	–	+	3/6
Su et al., 2017	M	7q11-qter; Mosaic	at term	–	+	1.910 (−3)	n.a.	n.a.	n.a	+	6.5	9.500 (−6)	91.9 (−6)	49 (−1)	+	+	–	–	3/6
Eggermann et al., 2008	F	7q11.2-qter	37	–	–	2.800 (−0.45)	46 (−1.16)	n.a.	n.a	+	5.3	n.a.	99.5 (−2.86)	n.a.	+	+	–	+	3/6
Eggermann et al., 2008	M	7q11.2-qter	37	–	+	2.180 (−2.28)	45 (−1.97)	32 (−1.34)	–	+	1.3	6.700 (−4.12)	73 (−3.6)	45 (−2.26)	+	–	–	+	3/6
Reboul et al., 2006	M	7q21-qter; Mosaic	27	+	+	600 (−3.5)	n.a.	n.a.	n.a	+	2.9	9400 (−3.5)	82 (−3.5)	47.5 (−2)	+	–	–	+	3/6
Hannula et al., 2001b	F	7q31-qter	37 + 5	+	+	1.510 (−4.3)	40 (−4.9)	n.a.	n.a	+	1.35	6.950 (−22%^*^)	71.5 (−2.9)	47 (−0.2)	+	+	–	+	4/6
Begemann et al., 2012	M	7q32-qter	39 + 5	+	+	2.410 (−2.74)	44 (−3.7)	32 (−2.77)	–	+	3.2	10.500	85.5 (−3.09)	46 (−3.51)	**–**	+	–	+	4/6
Total				3/7	5/7				0/3	7/7					6/7	5/7	0	6/7	

*GA, gestational age; IUGR, intrauterine growth restriction; SGA, small for gestational age; OFC, occipital frontal circumference; PNGF, post natal growth failure; Body asym, body asymmetry; SD, standard deviation;.n.a, not available*.

* *relative to median weight for height*.

**Table 2:** Comparative overview of the additional features in the present case and in the six reported cases with segmental upd(7q)mat.

**ADDITIONAL FEATURES**												
	**Sex**	**Disomic region extention**	**Triangular face**	**Fifth finger clinodactyly**	**Micrognathia**	**Low-set and/or posteriorly rotated ears**	**Down-turned mouth**	**High pitched/ squeaky voice**	**Speech delay**	**Irregular/ crowded teeth**	**Motor delay**	**Syndactyly of toes**
Present case	F	7q11.23-qter	−	+	–	+	–	+	+	–	–	+
Su et al., 2017	M	7q11-qter; Mosaic	+	+	n.a	–	–	–	–	+	+	–
Eggermann et al., 2008	F	7q11.2-qter	+	–	–	+	–	–	–	–	–	+
Eggermann et al., 2008	M	7q11.2-qter	+	+	+	+	+	–	–	–	+	–
Reboul et al., 2006	M	7q21-qter; Mosaic	–	–	–	–	–	–	–	–	–	–
Hannula et al., 2001b	F	7q31-qter	+	+	–	+	+	+	–	+	–	n.a
Begemann et al., 2012	M	7q32-qter	–	+	+	–	–	–	+	+	+	+
Total			4/7	5/7	2/7	4/7	2/7	2/7	2/7	3/7	3/7	3/7

*n.a, not available*.

In conclusion, this is the first report of uniparental isodisomy encompassing almost the whole long arm of chromosome 7 leading to PS and SRS-like features. While the inner ear phenotype of PS is always typical and the diagnosis easily achieved, the clinical features of the associated SRS are subtle and might have been undiagnosed in our case. The finding of a patient presenting without a relative macrocephaly at birth and without SGA, but with protruding forehead, postnatal growth failure, and feeding difficulties, usually accounted for by upd(7q)mat, recommends chromosome 7 investigation. In case of homozygous mutations inherited from one single carrier parent, such as in the case here presented, screening for upd is advisable.

## Author contributions

PF, SR, and LF conception and design. SM and MP provision of study materials or patients. VC, VG, CC, EM, AS, and MR collection and assembly of data. VC, VG, CC, EM, AS, MR, PF, SR, and LF data analysis and interpretation. VC, VG, CC, SM, EM, AS, MR, MP, DG, LL, LP, PF, SR, and LF manuscript writing. LL and LP final approval of manuscript. VC, VG, CC, SM, EM, AS, MR, MP, DG, LL, LP, PF, SR, and LF accountable for all aspects of the work.

### Conflict of interest statement

The authors declare that the research was conducted in the absence of any commercial or financial relationships that could be construed as a potential conflict of interest.

## Supplementary material

Supplemental Figure 1 Molecular-cytogenetic analyses on chromosomes X and CNV inheritance characterization **(A)** Identification in the patient of a 481 kb duplication at Xq26.2 (minimum interval chrX:130693373-131174432, GRCh37/hg19) using Agilent CGH 400K array, which partially included the *MST4* gene. The duplication was not maternal in origin. **(B)** qPCR analysis confirmed the presence of a duplication at Xq26.2 in the patient and documented its paternal origin. Cf, female control; Cm, male control; F, father; M, mother; P, patient.
