# Supplementary figures and images for: Segmental Maternal UPD of Chromosome 7q in a Patient With Pendred and Silver Russell Syndromes-Like Features
Source: Front Genet. 2018 Nov 30;9:600. doi: 10.3389/fgene.2018.00600 (PMC6284021; doi:10.3389/fgene.2018.00600)

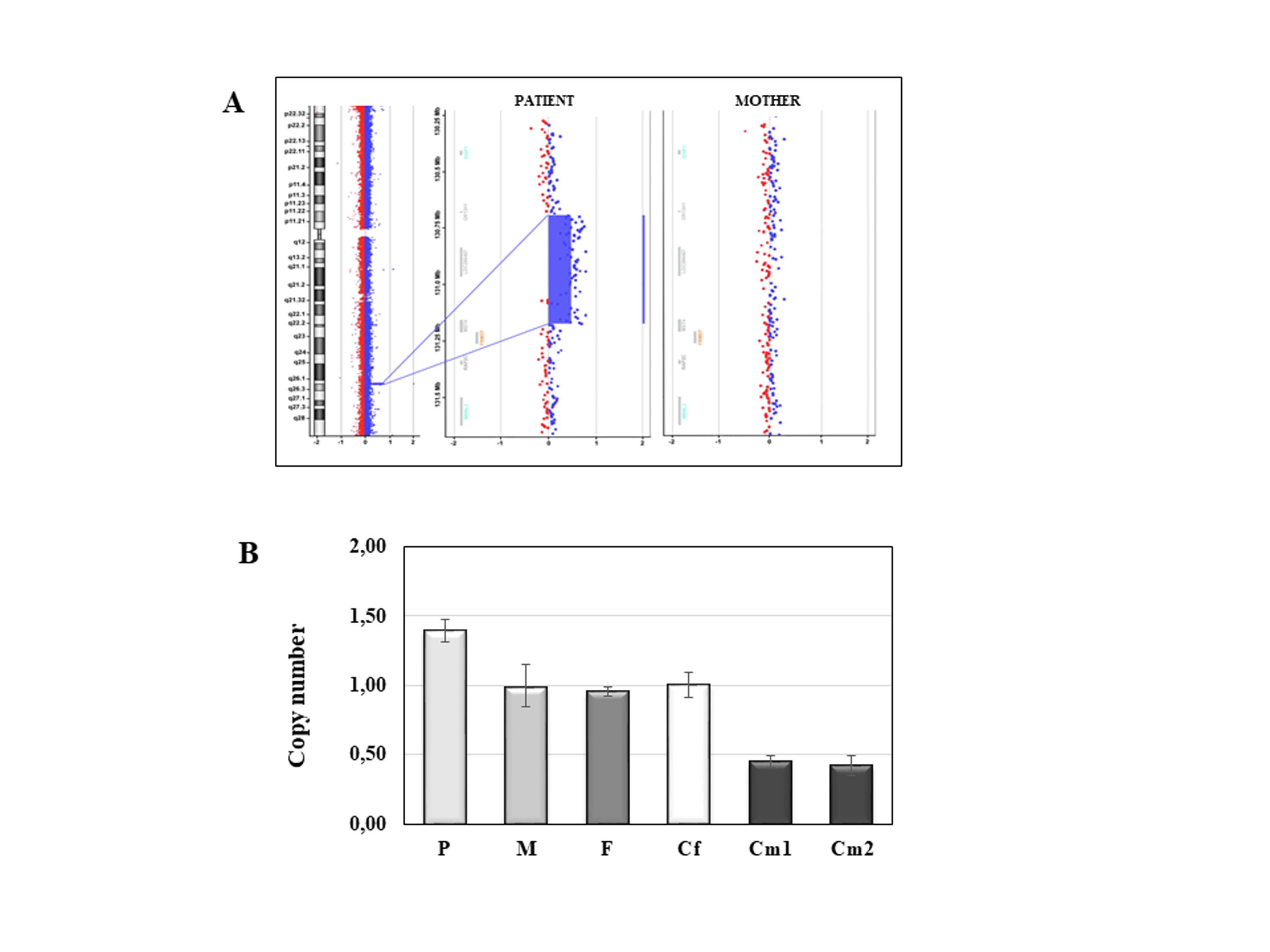

Supplement: Supplemental Figure 1 — Molecular-cytogenetic analyses on chromosomes X and CNV inheritance characterization (A) Identification in the patient of a 481 kb duplication at Xq26.2 (minimum interval chrX:130693373-131174432, GRCh37/hg19) using Agilent CGH 400K array, which partially included the MST4 gene. The duplication was not maternal in origin. (B) qPCR analysis confirmed the presence of a duplication at Xq26.2 in the patient and documented its paternal origin. Cf, female control; Cm, male control; F, father; M, mother; P, patient. [file Image_1.TIF]
